# Neural Network Model Based on Branch Architecture for the Quality Assurance of Volumetric Modulated Arc Therapy

**DOI:** 10.3390/bioengineering11040362

**Published:** 2024-04-11

**Authors:** Lizhang Xie, Lei Zhang, Ting Hu, Guangjun Li, Zhang Yi

**Affiliations:** 1 Machine Intelligence Laboratory, College of Computer Science, Sichuan University, Chengdu 610065, Chinazhangyi@scu.edu.cn (Z.Y.); 2 Cancer Center and State Key Laboratory of Biotherapy, Department of Radiation Oncology, West China Hospital, Sichuan University, Chengdu 610041, China

**Keywords:** branch architecture, quality assurance (QA), gamma passing rate (GPR), multi-branch neural network (MBNN), radiation therapy

## Abstract

Radiation therapy relies on quality assurance (QA) to verify dose delivery accuracy. However, current QA methods suffer from operation lag as well as inaccurate performance. Hence, to address these shortcomings, this paper proposes a QA neural network model based on branch architecture, which is based on the analysis of the category features of the QA complexity metrics. The designed branch network focuses on category features, which effectively improves the feature extraction capability for complexity metrics. The branch features extracted by the model are fused to predict the GPR for more accurate QA. The performance of the proposed method was validated on the collected dataset. The experiments show that the prediction performance of the model outperforms other QA methods; the average prediction errors for the test set are 2.12% (2%/2 mm), 1.69% (3%/2 mm), and 1.30% (3%/3 mm). Moreover, the results indicate that two-thirds of the validation samples’ model predictions perform better than the clinical evaluation results, suggesting that the proposed model can assist physicists in the clinic.

## 1. Introduction

In recent years, cancer mortality and incidence rates have been increasing year by year. Radiation therapy is an important cancer treatment method, and quality assurance (QA) is critical to ensuring the efficacy of the treatment and the safety of patients [1,2]. Patient-specific QA compares the measured and planned dose distribution using gamma analysis [3], including positional accuracy and dose accuracy. The general evaluation index for QA is the gamma passing rate (GPR) [4]. The GPR considers two types of error: dose deviation and distance to agreement. There are three combinations of dose deviation and distance-to-agreement commonly used for GPR: (2%/2 mm), (3%/2 mm), (3%/3 mm). 2% indicates that the dose deviation between measured and planned dose distributions within 2% is acceptable. A statement of 2 mm represents the distance between the measured and planned treatment point, whereby 2 mm is adequate. It can be seen that the error tolerance of the three sets of GPR increases successively. The GPR of the same plan will increase successively under the three sets. The value of the GPR ranges from 0% to 100%; when the GPR is closer to 100%, it means that the measured dose distribution is close to the planned dose distribution. The therapeutic effect of this plan is very good. Conversely, the closer the GPR is to 0%, the more unreliable the radiation therapy plan.

However, patient-specific QA measurement consumes a lot of time and effort for physicists, and this can lead to situations where it may be too late to react without this, resulting in treatment delays [5]. Due to resource constraints, patient-specific QA is difficult to complete. Most hospitals sample some treatment plans for patient-specific QA, and based on these sampling results, they evaluate for whole plans. If the GPR of the majority of samples is qualified, it indicates that the linear accelerator (Linac) is operating well, with stable performance and high treatment accuracy. On the contrary, if the sampling shows a low GPR and unqualified samples, it means that Linac is running in poor condition and needs to be corrected. Sampling QA uses as few resources as possible to ensure the overall effectiveness of treatment. However, there are still some unqualified radiation therapy plans that cannot be detected. Therefore, it is helpful to employ computer-aided diagnosis (CAD)-based QA methods to assist in patient-specific QA.

Since the complexity metrics of the Linac parameters and plan properties can affect the effect of radiation therapy, the CAD-based QA methods for intensity-modulated radiation therapy (IMRT) technology can achieve good performance by using machine learning methods to extract complexity metric features [5,6,7]. A novel IMRT known as volumetric modulated arc therapy (VMAT) was introduced by Otto et al. [8] in 2007. Thanks to the benefits of shortening treatment time, highly conformal dose distribution, and the expectation of toxicity limitation, VMAT became the commonly used and extremely important technique in radiation therapy, especially for head and neck (H&N), pelvis, and rectum tumors. However, VMAT is more complex, with more treatment control points and faster dose delivery. As a result, there are a greater number of complexity measures that affect QA, and they are more complex. The inefficient extraction of VMAT complexity metric features causes the IMRT-based QA approach to perform poorly in VMAT. Thus, it is imperative to propose CAD-based QA for VMAT technology.

Recently, the development of deep neural networks (DNNs) has created breakthroughs in various kinds of research because of their strong representation ability. They also have great significance implications for QA studies [9,10,11]. Despite having more powerful feature extraction capabilities, DNNs are still unsatisfactory in terms of reported performance. The QA performance enhancement effect is limited by relying only on the powerful feature extraction capability of DNNs. Neural network research on QA features adapted to VMAT is the way to enhance QA prediction performance.

Based on analyzing the features of the complexity metrics of VMAT, they can be classified into two different categories: one relates to the Linac, and the other relates to the plan properties. The two categories of complexity metrics work together to create the Linac error. The complexity metrics in the same category have strong intra-class dependencies between them, while the dependencies between complexity metrics in different categories are weak. While several studies have shown that branching networks are better for multi-category representation learning and feature fusion [12,13,14,15], no studies have focused on the features of VMAT complexity metric category features. Therefore, it is necessary to strengthen the intra-class feature extraction capability of the same category of complexity metrics, which is of great significance for the improvement of model performance.

In order to assist the patient-specific QA for VMAT technology, this paper proposes a neural network model based on the branch architecture for the QA of VMAT. The proposed neural network model mainly contains a branch feature extraction module and a multi-branch feature fusion module. The branch feature extraction module is designed with three branches for learning the features of the complexity metrics of Linac parameters, plan properties, and all complexity metrics, respectively. When compared to models without designed branches, different branches learn different categories of complexity metrics, which reduces the difficulty of learning complexity metrics within categories and makes it easier to achieve good learning results. The multi-branch feature fusion module merges the features extracted from the three branches for GPR prediction.

The main contributions of this paper can be summarized as follows:

1. By designing different branches to build the multi-branch network, it adopts different branches to learn different complexity metrics to predict the GPR. This brings two benefits: on the one hand, each branch focuses on learning intra-class features, and on the other hand, each branch needs to focus on fewer features. The branching design enhances the feature extraction capability of the model: each branch extracts different category complexity metric features, and these features are fused as a more comprehensive feature to the predictor for GPR prediction.
2. The prediction results contribute to improved clinical application. Samples with large errors between model predictions and clinical evaluations are revalidated. The results show that the model predictions of two-thirds of the validation samples outperform the clinical evaluation results, suggesting that the proposed model can assist physicists in the clinic.
3. A QA dataset of VMAT containing 850 samples with more than 10 cancers was constructed.

## 2. Related Work

With the rapid development of DNNs in recent years [16], there have been significant breakthroughs in computer vision, natural language processing, etc. This also aroused great interest among researchers and led to many related advances in the field of medicine [17]. Moreover, DNNs have made significant breakthroughs in radiation therapy [18,19], including treatment outcome prediction [20] and treatment optimization [21,22] et al. However, there are few DNN studies on QA, and the success of DNN methods in radiation therapy shows its strong potential in QA.

The essential task of the QA study is to predict the GPR, which is used to ensure the feasibility and safety of the treatment plan. The most reported QA studies are those that predict the GPR through the complexity metrics of the Linac parameters and plan properties [6,7,23]. Such studies extracted the complexity metrics of the Linac parameters and plan properties from the radiation therapy plan, preserving as much important information as possible and significantly reducing the number of parameters. For example, Gilmer et al. [6] used 498 IMRT plans to learn the characteristics of the plans through regression methods to predict the GPR. Tomohiro et al. [10] compared the GPR prediction performance among regression tree analysis (RTA), multiple regression analysis (MRA), and the DNN method. DNNs performed slightly better than RTA and MRA in terms of prediction error. Li et al. [7] used the Poisson lasso (PL) regression model to predict the GPR. Its performance at 3%/3 mm and 3%/2 mm is acceptable, but it is not acceptable at (2%/2 mm). Granville et al. [5] used SVM to predict the GPR, but the predicted outcomes were not prominent. However, they found that all complexity metrics are important in predicting outcomes.

Some studies try to reconstruct the flux map from the radiation therapy plan to predict the GPR [9,11]. The flux maps cover more parameters of therapy than the complexity metrics. However, the dataset of radiation therapy plans is usually very small, which makes it difficult for models to learn characteristics effectively. Yannet et al. [9] compared the performance of DNNs against a technique designed by domain experts in the prediction of the GPR for IMRT. The results showed that DNNs could achieve performance similar to that of a technique designed by domain experts. While DNNs are great for feature extraction, the small sample size of the dataset becomes a limitation to their performance. Noriyuki et al. [11] developed a CNN-based prediction model for the patient-specific QA of dose distribution in prostate treatment. The results suggested that deep learning may provide a useful prediction model for the gamma evaluation of patient-specific QA in prostate treatment planning. However, the performance of these studies is not enough to meet clinical needs, and QA may still lag behind the clinical treatment. Hu et al. [24] proposed the construction of a 3D convolutional neural network model using multimodal data. Fusing flux map features and dose features with its powerful feature extraction capability achieves good performance. However, it requires a large sample size, which is more difficult to collect.

Several studies show that branching networks are better for multi-category representation learning and feature fusion. Zhou et al. [12] proposed a bi-lateral-branch network (BBN), which consists of two branches: the conventional learning branch and the rebalancing branch; each branch performs its representation learning and classifier learning tasks. In the two-branch framework [13], the two branches maintain independent computational processes and receive different categories of data to co-learn. The bi-lateral segmentation network (BiSeNet) [14,15] consists of two branches: a spatial path network and a context path network, which are designed to respond to the loss of spatial information and the reduction in the sensory field, respectively. These studies indicate that decomposing the task into different branches makes the task simpler for each branch of the network. Hence, it is easier to train the model for good feature representation and pool these features for re-representation via feature fusion.

This paper aims to address the inefficiency of measuring QA and the poor performance of machine learning-based QA. A neural network model based on the branch architecture is proposed based on the complexity metrics of the Linac parameters and plan properties. It uses different branches to learn different categories of complexity metrics, leading to better feature extraction performance.

## 3. Materials and Methods

### 3.1. Dataset

The dataset for this study was collected at the West China Hospital of Sichuan University. All the VMAT plans were calculated and optimized with the Raystation treatment planning system (version 4.7, RaySearch Medical Laboratories AB, Stockholm, Sweden) with a 6 MV flattening filter. Treatment plans were delivered on Linac 1, Elekta Versa HD™ with the Agility multileaf collimators (Elekta, Crawley, UK), Linac 2, and Linac 3, Elekta Synergy Linac Systems with the Agility multileaf collimators (Elekta, Crawley, UK), respectively. The 6 MV photon beams of three Linacs were matched with the acceptance criterion that the difference in PDD10 among the matched Linacs was within ±1%, and any point dose within 80% of the FWHM region fell within a 2% different window for the flatness and symmetry of the beam profiles. The patient-specific QA was performed using the ArcCHECK detector array with a Cavity plug and chamber insert (Sun Nuclear Corporation, Melbourne, FL, USA), and it was analyzed using the SNC Patient software (version 6.7). The dose calculation algorithm for the VMAT plan was collapsed cone convolution (CCC, Raystation, version 4.7) with a calculational grid of 3.0 mm. The dose effect of the treatment couch was taken into account in the dose calculation. In accordance with the recommendations of the AAPM TG-218 report, gamma criteria of 3%/3 mm, 3%/2 mm, and 2%/2 mm with a 10% dose threshold, absolute dose mode, and global normalization were used for computing the gamma passing rate (GPR) by comparing the measured dose planes with the calculated dose planes.

The dataset used in this study contains 850 samples of radiation therapy plans, which were collected from the West China Hospital at Sichuan University. Table 1 shows the number of samples for each cancer in the dataset. While most datasets only focus on single cancers, ours covers the common cancers in the human body, including the abdomen, brain, breast, head and neck (H&N), nasopharyngeal carcinoma (Npc), pelvis, prostate, rectum, stomach, and others.

In this study, each sample contains 47 complexity metrics. According to the correlation between complexity metrics, they are divided into two categories: the Linac parameters and plan properties [25]. Table 2 shows the complexity metrics of the Linac parameters, including 25 complexity metrics in five categories. These complexity metrics describe the Linac state and settings during radiation therapy.

Next, we provide an explanation of complexity metrics.

* SASX mm: the percentage of small aperture score < X mm
* leaf gap X–Y mm: the percentage of leaf gap X mm < Y mm
* mean jawX gap: the average gap of jawX
* mean jawY gap: the average gap of jawY
* jawY gap 0–X mm: the percentage of jawY gap < X mm
* jawX gap 0–X mm: the percentage of jawX gap < X mm

Table 3 shows the 22 complexity metrics of the plan properties.

**Figure 1:**
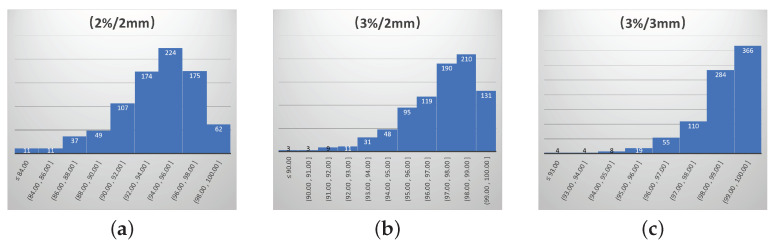
Distribution of GPRs in 850 radiation therapy plans. (**a**) The value distribution of the GPR at 2%/2 mm; (**b**) the value distribution of the GPR at 3%/2 mm; (**c**) the value distribution of the GPR at 3%/3 mm.

The distribution of GPR values for the 850 radiation therapy plans is shown in Figure 1. The GPRs of 2%/2 mm, 3%/2 mm, and 3%/3 mm are in the range of [78.90–100.00%], [89.00–100.00%], [91.10–100.00%], respectively. The GPRs of the same samples measured under different error tolerability standards are different. The higher the tolerability of the measurement error, the higher the value of the GPR. Among the three sets of GPRs, the criterion of 2%/2 mm is the most important. It has the lowest tolerability for error and is the most important criterion in clinical evaluation, which can provide the best accuracy measurement of the radiation therapy plan. Therefore, this paper focuses on the 2%/2 mm gamma criterion for GPR prediction.

The American Association of Physicists in Medicine TG 218 report [26] recommended 95% and 90% as the tolerability and action limits for the 3%/2 mm gamma criterion, respectively. The report does not recommend tolerability and action limits for 2%/2 mm and 3%/3 mm. Therefore, by comparing the sample number of 3%/2 mm, the tolerability and action limits for 2%/2 mm were set at 90% and 84%, and the tolerability and action limits for 3%/3 mm were set at 97% and 93%. The tolerability and action limits for the three VMAT gamma standards are shown in Table 4.

### 3.2. The Neural Network Model Based on Branch Architecture

**Figure 2:**
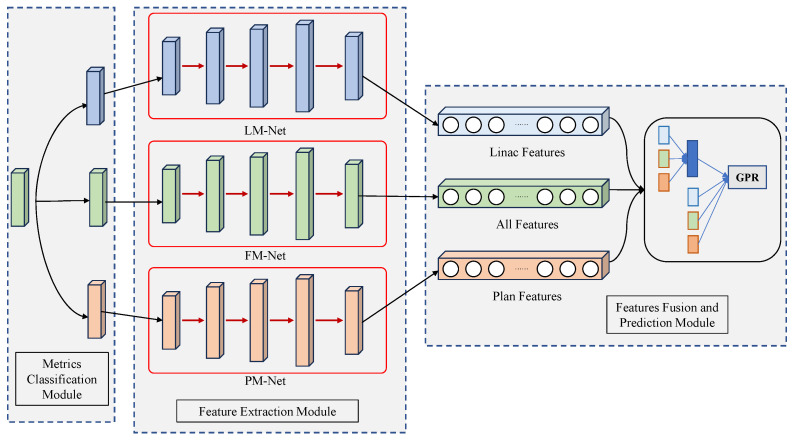
The proposed multi-branch neural network model.

The details of the proposed neural network model based on branch architecture are shown in Figure 2, which is named the multi-branch neural network (MBNN). The proposed MBNN model framework contains three modules: the metric classification module, the feature extraction module, and the feature fusion and prediction module. All the complexity metrics were used as the model inputs and are classified into two categories by the metrics classification module. The different categories of the metrics are fed into the feature extraction module, which contains three branches for extracting the features of different categories of metrics. Three branches are defined as full metrics networks (FM-Nets), Linac metrics networks (LM-Nets), and plan metrics networks (PM-Nets), respectively. The features extracted by the feature extraction module are pooled into the feature fusion and prediction module for feature fusion and the prediction of the GPR. The branching design enhances the feature extraction capability of the model: each branch extracts different category complexity metric features, and these features are fused as a more comprehensive feature to the predictor for GPR prediction.

The data used in this paper contain the complexity metrics and corresponding measured GPRs $Q=\{\left(x_{n},y_{n}\right);n\in \left(1,2,\cdots ,N\right)\}$, where $x_{n}$ represents the complexity metrics, and $y_{n}$ is the GPR of the *i*-th sample, respectively. For the complexity metrics of sample $x_{n}$, the complexity metrics of the Linac parameters and plan properties are defined as $x_{n}^{lm}$ and $x_{n}^{pm}$, respectively.

The GPR prediction study in this paper is formally denoted as

(1) $$F:x_{n}\to p_{n},$$

where *F* is the QA prediction study, and $p_{n}$ is the predicted GPR of $x_{n}$. The value of the GPR range is from 0% to 100%.

Specifically, the formulas of the three branch networks are defined as follows:

(2) $$\begin{array}{cc} \; & f_{LM}:x_{n}^{lm}\to f_{n}^{lm},\\ \; & f_{PM}:x_{n}^{pm}\to f_{n}^{pm},\\ \; & f_{FM}:x_{n}\to f_{n}^{fm}, \end{array}$$

where $f_{FM}$, $f_{LM}$, and $f_{PM}$ are the FM-Net, LM-Net, and PM-Net, respectively. $f_{n}^{lm}$, $f_{n}^{pm}$ and $f_{n}^{fm}$ are the features of LM-Net, PM-Net, and FM-Net, respectively.

Each branch network consists of two parts: the input layer and the feature extraction layer. The input layer is used to receive the complexity metrics of each sample. Each sample has 47 complexity metrics, including 25 complexity metrics for the Linac parameters and 22 complexity metrics for the plan properties, so the number of neurons in the input layer of FM-Net, LM-Net, and PM-Net are 47, 25, and 22, respectively. The feature extraction layer is designed as three fully connected layers. The number of neurons in the fully connected layers in FM-Net are 128, 512, and 128, respectively. The number of  neurons in the fully connected layers in LM-Net and PM-Net are 64, 256, and 64, respectively. For the activation functions of the neural network, except for the last layer, which uses sigmoid, the rest of the neural network uses the relu activation function.

In the multi-branch feature fusion module, the features extracted from the three branch networks are merged to receive the fused features:

(3) $$f_{n}^{fuse}=\left[f_{n}^{lm},f_{n}^{pm},f_{n}^{fm}\right].$$

The prediction of each branch has an impact on the final prediction, and the formula of the final prediction is defined in detail as follows:

(4) $$F:f_{n}^{fm}\times w_{fm}+f_{n}^{lm}\times w_{lm}+f_{n}^{pm}\times w_{pm}+f_{n}^{fuse}\times w_{fuse}\to p_{n},$$

where $w_{fm}$, $w_{lm}$, $w_{pm}$, and $w_{fuse}$ are the weights of the features of LM-Net, PM-Net, FM-Net, and the fused features, respectively. They are set to 0.3, 0.15, 0.15, and 0.4, respectively.

The loss function of the proposed model is

(5) $$L=l_{FM}+\lambda 1\times l_{LM}+\lambda 2\times l_{PM},$$

where *L* is the loss function of the proposed model, and it consists of three loss functions. $l_{LM}$, $l_{PM}$, and $l_{FM}$ are the loss functions of the LM-Net, PM-Net, and FM-Net, respectively. λ1 and λ2 are set as 0.5. $l_{LM}$, $l_{PM}$, and $l_{FM}$ are defined as follows:

(6) $$\begin{array}{cc} \; & l_{FM}=\frac{1}{N}\sum_{n=1}^{N}{\left(y_{n}-p_{n}\right)}^{2},\\ \; & l_{PM}\&l_{LM}=\frac{1}{N}\sum_{n=1}^{N}\left(y_{n}-p_{n}\right), \end{array}$$

where $y_{n}$ and $p_{n}$ are the true and predicted GPRs, respectively. The mean squared error (MSE) is the common loss function; hence, it is used for PM-Net, which contains all the complexity metrics. The mean absolute error (MAE) measures the distance between the predicted and true GPR and is used for branch network evaluation with fewer complexity metrics.

The overall learning process of the proposed multi-branch neural networks is shown in Algorithm 1.
**Algorithm 1** Framework of multi-branch neural networks model**Input:** The complexity metrics $x_{n}$($n\in \left(1,2,\cdots ,N\right)$)**Output:** The prediction $p_{n}$ of input $x_{n}$($n\in \left(1,2,\cdots ,N\right)$)
  1:Ending epochs = 200  2:Initializing the model randomly  3:**while** training epoch < ending epochs **do**  4:   **for** a case $x_{n}$ in dataset Q **do**  5:     $f_{LM}:x_{n}^{lm}\to f_{n}^{lm}$  6:     $f_{PM}:x_{n}^{pm}\to f_{n}^{pm}$  7:     $f_{FM}:x_{n}\to f_{n}^{fm}$  8:     $f_{n}^{fuse}=\left[f_{n}^{lm},f_{n}^{pm},f_{n}^{fm}\right]$  9:     Prediction: $f_{n}^{fm}\times w_{fm}+f_{n}^{lm}\times w_{lm}+f_{n}^{pm}\times w_{pm}+f_{n}^{fuse}\times w_{fuse}\to p_{n}$10:     Updating gradients with back propagation algorithm11:   **end for**12:**end while**13:**while** training epochs = ending epochs **do**14:   Saving the model and parameters15:**end while**

## 4. Experiments

### 4.1. The Experimental Setup

A total of 850 samples are collected in our dataset, which is randomly split into training and test sets according to the ratio of 4:1 in this paper. The training set includes 680 samples, while the test set includes 170 samples. Each sample contains 47 complexity metrics and three GPRs. Each GPR of the same sample is measured with different dose deviation/distance-to-agreement criteria. All experiments were conducted on this dataset.

The proposed model takes 47 complexity metrics as input, 45 of them are numerical data, which are preprocessed by standardization, and two non-numeric complexity metrics are encoded by one-hot. The mean square error (MSE) is used as the cost function, as shown in Equation (3). The optimizer is sgd with a learning rate of $1\times 10^{-3}$. The learning rate decays by 0.98 every five epochs. The batch size is 200. In order to reduce the side effects of overfitting, dropout is applied in the last hidden layer with a probability of 0.6. The mean absolute error (MAE) is used to evaluate our model, which visually describes the distance between the measured and predicted GPR. It is the most important criterion for clinical radiologists. The calculation of MAE is presented as follows:

(7) $$MAE=\frac{1}{N}\sum_{n=1}^{N}\left|y_{n}-p_{n}\right|.$$

### 4.2. Results

As mentioned above, QA studies on VMAT are mostly based on traditional machine learning methods, including SVM [5] and lasso regression [7]. Moreover, some common machine learning methods, such as RF and k-nearest-neighbor (KNN), are incorporated. The DNN method contains 3D-MResNet [24] and ONO-Net [10]. The proposed method is compared with other reported state-of-the-art methods. The experimental results of the test set are shown in Table 5. It can be seen that the proposed method achieves the best performance on all GPR predictions, obtaining an MAE of 2.12% at 2%/2 mm, 1.69% at 3%/2 mm, and 1.30% at 3%/3 mm. Due to the distribution of the data, the MAE results show no apparent differences among all methods at 3%/3 mm, which has no great importance for the clinical assistance as expected. The proposed method is significantly better than other machine learning methods for the 2%/2 mm and 3%/2 mm gamma criteria. In particular, the MAE of the proposed method is 15–23% lower than that of other methods on the most important clinical evaluation criterion: 2%/2 mm. It takes 15 s for the model to train one epoch. Meanwhile, the performance of the proposed model is better than that of ONO-Net [10], which is a DNN without a branch design. This shows that our branch network design has a significant impact on QA performance. The 3D-MResNet [24] model performs well, but it requires additional dose features.

**Figure 3:**
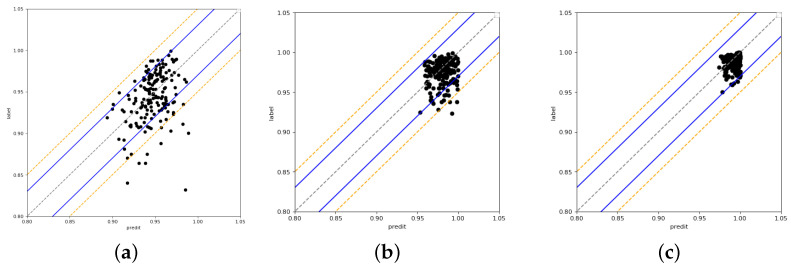
The predicted GPR and measured GPR of three gamma criteria. (**a**) 2%/2 mm; (**b**) 3%/2 mm; (**c**) 3%/3 mm.

The predicted and measured GPRs for the test set are displayed in Figure 3. Each point represents a sample, and the points enclosed by the blue and orange lines show that the sample’s MAE is <3% and <5%, respectively.

The results of the 2%/2 mm gamma criterion are displayed in Figure 3a, where the majority of the samples have prediction errors of <5%. There are 170 samples in the test set overall, of which 159 have prediction errors of <5% and 11 have prediction errors of >5%. However, one sample has a very large prediction error of 15%, which can not be accepted. Figure 3b shows the results of the 3%/2 mm gamma criterion, where 90% of the samples had a prediction error of <5%. Five samples had a prediction error of >5%. When compared to the 2%/2 mm gamma criterion, the samples are more centered on the midline, which means that the prediction error is smaller. This prediction error performance is valuable for assisting in patient-specific QA. Figure 3c shows the results of the 3%/3 mm gamma criterion, and the prediction errors are basically <3%. Only a few samples have prediction errors of >3%

The experimental results demonstrate that the prediction of our model is very good, especially at 3%/2 mm. The American Association of Physicists in Medicine TG 218 report recommends 95% and 90% as the tolerability and action limits for 3%/2 mm, respectively. At 3%/2 mm, most of the predicted GPRs are >94%, and even when taking a 3% prediction error into account, the predictions of our model meet the action limit. The tolerability and action limits for 3%/3 mm are set as 97% and 93%. Most of the predicted GPRs are >96%, taking into account a 3% prediction error; thus, the predictions of our model meet the action limit. The result indicates that the prediction performance of the proposed multi-branch neural network QA model is good. However, a few samples with large prediction errors for the 2%/2 mm gamma criterion will lead to model unavailability.

**Figure 4:**
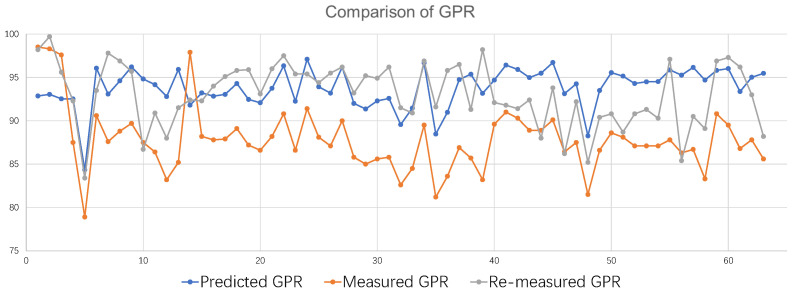
A comparison of the GPRs.

There is a huge deviation between the predicted GPR and the measured GPR at 2%/2 mm for all 63 samples during the experiment, with a total of 52 from the training set and 11 from the test set. Errors in the test set are reasonable, but the training set should not have such large errors. After a discussion with the physicists, one possible explanation is that the errors occurred in the patient-specific QA measurement. That means the measured GPRs of these samples may be inaccurate. These 63 samples were re-measured for patient-specific QA. A comparison of the first measurement, re-measurement, and model-predicted GPRs is shown in Figure 4. The blue line is the model-predicted GPR, the orange line is the GPR for the first patient-specific QA measurement, and the gray line is the GPR for the patient-specific QA re-measurement. The re-measurement of the GPR was used as a benchmark for comparison. The samples from the first measurement had an overall low GPR, which may be due to inaccurate Linac or operational errors. The re-measured GPR for two-thirds of the samples are closer to the model-predicted GPR, and the re-measurement GPRs of the rest of the samples is closer to the first GPR measurement. It suggests that the model-predicted GPR is closer to the true GPR. It also illustrates the inaccuracy of the first measurement of the GPR. This result further supports the model’s prediction performance, and the results of the model’s evaluation of inaccurately measured samples will be valuable to radiation therapy physicists.

**Figure 5:**
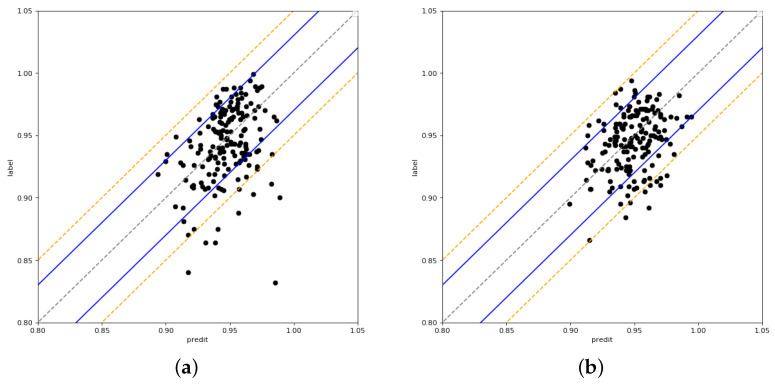
The predicted and measured GPRs. (**a**) (2%/2 mm); (**b**) Remeasured results of (2%/2 mm).

The model was re-trained by updating the dataset by replacing the first measured GPR in the 63 samples with the re-measured GPR. The MAE decreased from 2.12% to 1.92% for 2%/2 mm in the re-trained model. Figure 5b shows the predicted results of the test set. At 2%/2 mm, the prediction errors are mostly >5%. There is a significant decrease in the prediction error compared to the results of the first measurement in Figure 5a. In the new dataset especially, there are almost no samples with large prediction errors. The number of samples with prediction errors of >5% decreased from 11 to 8. The maximum prediction error of the samples decreased from 15% to 8%. The number of samples with a deviation of >5% both in the training set and test set decreased from 63 to 35. The number of samples in the test set with a prediction error of >5% decreased significantly, from 52 to 27. The tolerability and action limits for 2%/2 mm are set as 90% and 84%. Most of the predicted GPRs are >90%, taking into account a 5% prediction error; thus, the predictions of our model meet the action limit.

This shows that the proposed multi-branch neural network performs well. The model predictions have low mean errors, and there are no samples with significant prediction errors, which can assist in clinical QA in practice, hopefully optimizing the workflow of patient-specific QA.

### 4.3. Ablation Experiment

This section explores the number of layers and neurons in the hidden layer. The experiments were performed on the most important branch: FM-Net. As shown in Table 6, the 128-512-128 setting achieved the best performance.

Since the QA complexity metrics are distinctly different in the two categories, LM-Net and PM-Net are designed to learn the features of the two categories, respectively, and FM-Net is designed to learn the features of all the complexity metrics. The proposed model can learn the features of the complexity metrics with limited samples easier and better. Table 7 shows that the multi-branch networks outperform all single-branch networks, which demonstrates the success of the multi-branch networks in the QA study. It is worth noting that each branch network can predict the GPR independently, but the performance of single-branch networks is not as good as multi-branch networks.

LM-Net and PM-Net use fewer complexity metrics for prediction and, thus, do not perform as well as the multi-branch networks. At the same time, neither of these networks performs as well as FM-Net. This suggests that complexity metrics are important for feature representation, and more complexity metrics lead to better performance. The performance of FM-Net and ONO-Net is comparable. They both take all the complexity metrics as input, but the performance of ONO-Net is inferior to the proposed method. This is further evidence that the proposed method can learn features to predict the GPR better than a single network.

## 5. Conclusions

Patient-specific QA may lag behind treatment, and the performance of machine learning-based QA is not sufficient to assist patient-specific QA, all of which fail to ensure the dose delivery and safety of the patient. By analyzing the complexity metrics of the Linac parameters and plan properties of VMAT, a real-time multi-branch network is proposed for the QA of VMAT for multiple cancers. Our model can balance performance and efficiency, which effectively addresses the shortcomings of poor timeliness in patient-specific QA and the insufficient performance of machine learning-based QA. The experiments show that the proposed method is superior to other state-of-the-art machine learning methods. Moreover, the proposed QA model detected some samples with measurement GPR errors, which can assist physicists in performing patient-specific QA.

Furthermore, the performance of machine learning-based QA methods is not comparable to patient-specific QA, which is intended to support rather than replace patient-specific QA. The QA model can improve the workflow of radiation therapy physicists. It can evaluate the radiation therapy plan in real time when the plan is developed, and then patient-specific QA can be performed in time for those with low GPRs. This workflow ensures that all radiation therapy plans are evaluated by the QA model, and the possibility of unqualified samples being missed is reduced while not taking up medical resources. This is important for treatment effectiveness and patient safety.

For future research, it would be promising to extend the current work in the following areas: (1) More complexity metrics can be mined from the VMAT radiation therapy plan for GPR prediction. (2) It is possible to analyze the sensitivity of the complexity metrics for the GPR predictions of different cancers. (3) The flux map reconstructed from the VMAT plan can be used to predict the GPR when a much larger set of samples is collected.

## Figures and Tables

**Table 1:** Number of cancer samples in the dataset.

Cancer	Sample Number
Abdomen	80
Brain	28
Breast	4
H&N	117
Npc	127
Pelvis	162
Prostate	56
Rectum	186
Stomach	42
Other	48
Total	850

**Table 2:** The complexity metrics of the Linac parameters of the cancer samples in the dataset.

Category	Complexity Metrics
SASX mm	SAS2 mm, SAS5 mm, SAS10 mm, SAS15 mm, SAS20 mm, SAS30 mm
leaf gap X-Y mm	leaf gap 2–5 mm, leaf gap 5–10 mm, leaf gap 10–15 mm, leaf gap 15–20 mm, leaf gap 20–30 mm
mean jawi gap	mean jawY gap, mean jawX gap
jawY gap 0–X mm	jawY gap 0–2 mm, jawY gap 0–5 mm, jawY gap 0–10 mm, jawY gap 0–15 mm, jawY gap 0–20 mm, jawY gap 0–30 mm
jawX gap 0–X mm	jawX gap 0–2 mm, jawX gap 0–5 mm, jawX gap 0–10 mm, jawX gap 0–15 mm, jawX gap 0–20 mm, jawX gap 0–30 mm

**Table 3:** The complexity metrics of plan properties.

Complexity Metrics	Definition
Fraction dose	The fraction dose
Mean CP number	Mean control point number
Mean CP MU	Mean control point monitor
PMU	Plan normalized MU
Beam number	The beam number
Linac	Linear accelerator
CAS	Cross-axis score
CIAO	Complete irradiated area outline
MAD	Mean asymmetry distance
PA	Plan area
PI	Plan averaged beam irregularity
PM	Plan averaged beam modulation
PALG	Plan average leaf gap
ALT X1	Averaged leaf gap of xl
ALT X2	Averaged leaf gap of x2
ALG	Averaged leaf gap
MCS	Modulation complex score
Doctor	The treating physicist
Positions	The treating position
MU1	MU value in first arc
MU2	MU value in second arc
TMU	Total MU

**Table 4:** The tolerability and action limits for the three gamma criterions of VMAT.

Gamma Criterion	Action Limit	Tolerability Limit
(2%/2 mm)	90%	84%
(3%/2 mm)	95%	90%
(3%/3 mm)	97%	93%

**Table 5:** The proposed method compared with state-of-the-art methods.

Method	MAE		
	(2%/2 mm)	(3%/2 mm)	(3%/3 mm)
The proposed MBNN	2.12%	1.69%	1.30%
SVM [5]	2.49%	1.95%	1.33%
RF	2.56%	1.90%	1.35%
KNN	2.78%	1.87%	1.34%
lasso regression [7]	2.60%	1.89%	1.36%
ONO-Net [10]	2.30%	1.77%	1.30%
3D-MResNet [24]	2.20%	1.73%	1.30%

**Table 6:** The setting of the hidden layers.

Hidden Layers	MAE of 2%/2 mm
128-512-1024-512-128	2.31%
128-1024-256-64	2.26%
128-512-128	2.23%
128-1024-128	2.31%
64-256-64	2.25%
32-128-32	2.39%
512-64	2.28%
256-64	2.27%

**Table 7:** Performance of the branch networks.

Method	MAE		
	(2%/2 mm)	(3%/2 mm)	(3%/3 mm)
Our method	2.12%	1.69%	1.30%
FM-Net	2.23%	1.70%	1.32%
LM-Net	2.67%	1.72%	1.52%
PM-Net	2.36%	1.90%	1.37%
ONO-Net	2.30%	1.77%	1.30%

## Data Availability

The data presented in this study are available upon request from the corresponding author.
